# Sex ratio at birth in Vietnam among six subnational regions during 1980–2050, estimation and probabilistic projection using a Bayesian hierarchical time series model with 2.9 million birth records

**DOI:** 10.1371/journal.pone.0253721

**Published:** 2021-07-14

**Authors:** Fengqing Chao, Christophe Z. Guilmoto, Hernando Ombao

**Affiliations:** 1 Statistics Program, Computer, Electrical and Mathematical Sciences and Engineering Division, King Abdullah University of Science and Technology, Thuwal, Saudi Arabia; 2 CEPED/IRD, Centre de Sciences Humaines, New Delhi, India; University of Western Australia, AUSTRALIA

## Abstract

The sex ratio at birth (SRB, i.e., the ratio of male to female births) in Vietnam has been imbalanced since the 2000s. Previous studies have revealed a rapid increase in the SRB over the past 15 years and the presence of important variations across regions. More recent studies suggested that the nation’s SRB may have plateaued during the 2010s. Given the lack of exhaustive birth registration data in Vietnam, it is necessary to estimate and project levels and trends in the regional SRBs in Vietnam based on a reproducible statistical approach. We compiled an extensive database on regional Vietnam SRBs based on all publicly available surveys and censuses and used a Bayesian hierarchical time series mixture model to estimate and project SRB in Vietnam by region from 1980 to 2050. The Bayesian model incorporates the uncertainties from the observations and year-by-year natural fluctuation. It includes a binary parameter to detect the existence of sex ratio transitions among Vietnamese regions. Furthermore, we model the SRB imbalance using a trapezoid function to capture the increase, stagnation, and decrease of the sex ratio transition by Vietnamese regions. The model results show that four out of six Vietnamese regions, namely, Northern Midlands and Mountain Areas, Northern Central and Central Coastal Areas, Red River Delta, and South East, have existing sex imbalances at birth. The rise in SRB in the Red River Delta was the fastest, as it took only 12 years and was more pronounced, with the SRB reaching the local maximum of 1.146 with a 95% credible interval (1.129, 1.163) in 2013. The model projections suggest that the current decade will record a sustained decline in sex imbalances at birth, and the SRB should be back to the national SRB baseline level of 1.06 in all regions by the mid-2030s.

## Introduction

The sex ratio at birth (SRB, i.e., the ratio of the number of male to female live births) varies between 1.03 and 1.07 under natural circumstances [1, 2]; however, in recent decades, the combined effects of the preference for a male child due to social, cultural, political, and economic reasons; fertility decline; and accessible and affordable abortion and sex detection technology have led to sex-selective abortion in several countries. Prenatal sex selection has resulted in distorted levels of the SRB in various South Asian, East Asian, and East European countries where SRBs can be as high as 1.1–1.2 male births per female birth [3, 4]. Additionally, several countries in South Asia and elsewhere where a son’s preference prevails may witness rising SRBs in the future as average fertility levels decline [5]. Not only is sex selection an obvious manifestation of the strength of gender bias, but it has also been projected that there will be tens of millions of excess adult men in affected countries after three decades [4].

Vietnam’s case has long drawn the attention of observers, as no increase in the SRB was witnessed until the beginning of the century—at a time when the SRB in nearby China was already close to a record 1.2 male births per female birth. The census and survey data failed to provide evidence of any distinct sex imbalances at birth [6, 7] despite several facilitating preconditions of prenatal sex selection such as the steady reduction of birth rates, emergence of modern private clinics, free and unrestricted access to abortion services, and strongly entrenched preference for a male child [8].

Signs of an increase in the SRB were, however, identified from the data collected by the nationally representative annual demographic sample surveys during the 2000s and finally confirmed by the exhaustive data from the 2009 census [5, 9–11]; these studies confirmed the sudden increase in the proportion of male births after 2005. Further studies documented a continued rise in the SRB that reached levels above 1.1 during the following decade [12]. More recent estimates point to a stabilization of SRB levels at the national level, i.e., close to 1.1–1.2, a feature corroborated by the results of the 2019 population census that put the SRB at 1.115 during the preceding year [13, 14]. The current stabilization of the SRB observed in Vietnam corresponds to the plateau level of the sex ratio transition—a transitional process characterized by a succession of initial rise, subsequent stabilization, and ultimate return to normalcy of the SRB [8]. This cycle has already been observed in several countries of East Asia or Eastern Europe such as South Korea, Georgia, Armenia, Azerbaijan, and China where the SRB started rising more than a decade earlier than in Vietnam [2]. The stabilization of the SRB and its potential turnaround in the years to come might also result from the concerted action by the Vietnamese government against the spread of sex-selective practices through different campaigns and policy initiatives such as the 2006 Law on Gender Equity, the 2011–2020 Strategy on Reproductive Health, and the regulations of the health sector introduced in 2013 and 2014, even if their real effectiveness remains undetermined [15, 16].

A distinctive feature of sex imbalances at birth in Vietnam lies in their uneven geographic distribution as measured by estimates by the administrative division, i.e., Vietnam’s six macro-regions in this study. The analysis of birth data demonstrated that the SRB ranges from almost normal levels in the regions of the south while it reaches levels well above the national average in the north, especially in the region of the Red River Delta. According to the 2019 census estimates, the SRB ranges from levels around 1.05 in the southern provinces to levels above 1.18 in several provinces close to Hanoi. In other words, there are areas in Vietnam without sex imbalances at birth, while in the northern provinces, the SRB can be distinctly higher than the world’s record—currently in Azerbaijan with an estimated SRB estimated at 1.14 in 2019 [17]. These regional differences are related to variations in fertility levels but also to cultural and socioeconomic differences and their impact on the intensity for the preference of a male child [12]. To understand the evolution of sex imbalances at birth in Vietnam in the future, it is therefore of primary importance to disaggregate the analysis by regional unit.

Previous studies utilized Bayesian methods for estimating [2] and projecting [5] the SRB at a national level for all countries from 1950 to 2100, including Vietnam. However, the levels and trends in SRB for the entire country conceal the variations within the country. For a country of demographic and cultural heterogeneity like Vietnam, it is essential to model the SRB on a subnational level and test whether the SRBs in the northern provinces deviate from those in the rest of the country. However, estimating Vietnam’s SRB is fraught with challenges due to the diversity of available sources (census and sample survey data), inconsistency of reported levels and trends derived from these data sources, and lack of reliable registration data on birth [13, 18]. The data issues are exacerbated at the subnational level as the data quality is more fragile compared to at the national level due to the smaller size of regional samples, considering that the quality of SRB estimation is closely related to the number of sample births used [19]. The paucity of data hinders the complete understanding or regional sex ratio transition (e.g., when and where the turnaround in SRB levels may occur), and this aspect explains why no attempt has been made so far to develop projections of the SRB by region in Vietnam.

Given these reasons, the analysis of the dynamics of sex imbalances at birth in Vietnam requires the estimation and projection of regional SRB levels and trends based on the largest data set possible and on a reproducible statistical model. In this study, we estimate Vietnam’s SRBs by subnational region from 1980 to 2019 and project them till 2050 based on a Bayesian hierarchical time series mixture model (hereafter, we use the term “region” to refer to the subnational regions of Vietnam). The Bayesian model uses all available data and synthesizes the differences in the levels and trends across data sources in a statistical and reproducible fashion. We first compiled a comprehensive database on regional observations of births by sex from nationally representative surveys and censuses in Vietnam. We then modeled the sex ratio transition and identified Vietnamese regions with skewed SRB levels.

### Social, demographic and cultural determinants of high SRB in Vietnam

When analyzing the rise of sex-selective abortions in Vietnam, it is important to underline the role of three factors also found in the other countries from Eastern Europe to South Asia where the SRB has risen from the 1980s onward [8]. The preconditions associated with skewed SRB levels relate to the supply factor (access to sex selection technology, i.e. prenatal diagnosis and abortion), the demand factor (the perceived need for a male child), and the squeeze factor (the higher risk of sonlessness caused by declining fertility). These different factors have clearly been at play in Vietnam.

Starting with squeeze factor, fertility has indeed declined rapidly in the country over the last forty years and is now close to replacement level at 2.1 children per woman (as per the 2019 census). The probability to have a male child has therefore declined and 22% of parents may end up sonless today in the absence of deliberate sex selection. This fertility decline is partly due to the family planning policies introduced by the government in the 1990s. It is, however, worth stressing that policies to encourage Vietnamese women to have no more than 2 children have never been as stringent as birth policies enforced in China after 1980. This may partly explain why the SRB started rising in Vietnam rather late compared to China, South Korea, or India where it occurred twenty years earlier.

The change in the supply factor accompanying rising SRBs is no such linked not to abortion–which has been long allowed in Vietnam and is frequently used by parents to limit family size–as to the appearance prenatal diagnosis. The emergence of reliable and affordable prenatal screening techniques dates only to the beginning of the 21st century [20–22]. This relative delay also explains the gap with China and other countries where the rise of the SRB took place earlier. While now officially forbidden, the disclosure of the sex of the fetus appears quite common and most parents know the sex of their children before birth. Initial policy measures to encourage ultrasound testing may have unintentionally facilitated the diffusion of sex-selective abortions. The booming private health sector has drawn large revenues from the sustained demand from pregnant women for prenatal diagnosis and abortion services.

The determining factor in the equation remains, however, son preference [23–25]. Its origin can be attributed to the Chinese influence in the north of the country, which China rules till the 10th century. They left behind a social and political system deeply influenced by the Confucian traditions, which is at the root of the strong gender bias and male domination. According to these patriarchal traditions, men control their daughters, their sisters, their wives, and their mothers after the death of the father. The most patent form of gender bias is the patrilocal co-residence system, whereby newly married couples stay with the husband’s family. On marriage, women lose their native family’s identity and join the patrilineage of their spouse. Producing a son is necessary for the perpetuation of the family line and will enhance the status of the daughter-in-lay in the family. Families without sons are looked down upon. The family name as well as family property, most notably agricultural land, will be transmitted only to sons, who are supposed to take care of their parents in old age as well as to continue the ancestors’ worship after their parents’ death.

Contemporary social and economic change has not yet suppressed son preference, which remains closely linked to the patrilineal and patrilocal kinship system found in Vietnam, especially in the North and among the ethnic *Kinhs*. The family system (patrilocality, exclusion of daughters from inheritance), economic benefits (old age support and social protection offered by male children) and traditional attitudes (ancestor worship by sons, low reputation of sonless couples) represent strong incentives for parents to have at least a son even among urban middle-class families that do not rely anymore on land and may not be deeply religious. The role of gender norms that are skewed in favor of boys is almost as important as the actual economic benefits that parents might draw from their sons, since married daughters may also be relied upon for support [15]. But the projected turnaround of the SRB trends announces the gradual erosion of son preference across the country in the coming decades.

## Materials and methods

### Region grouping

**Fig 1:**
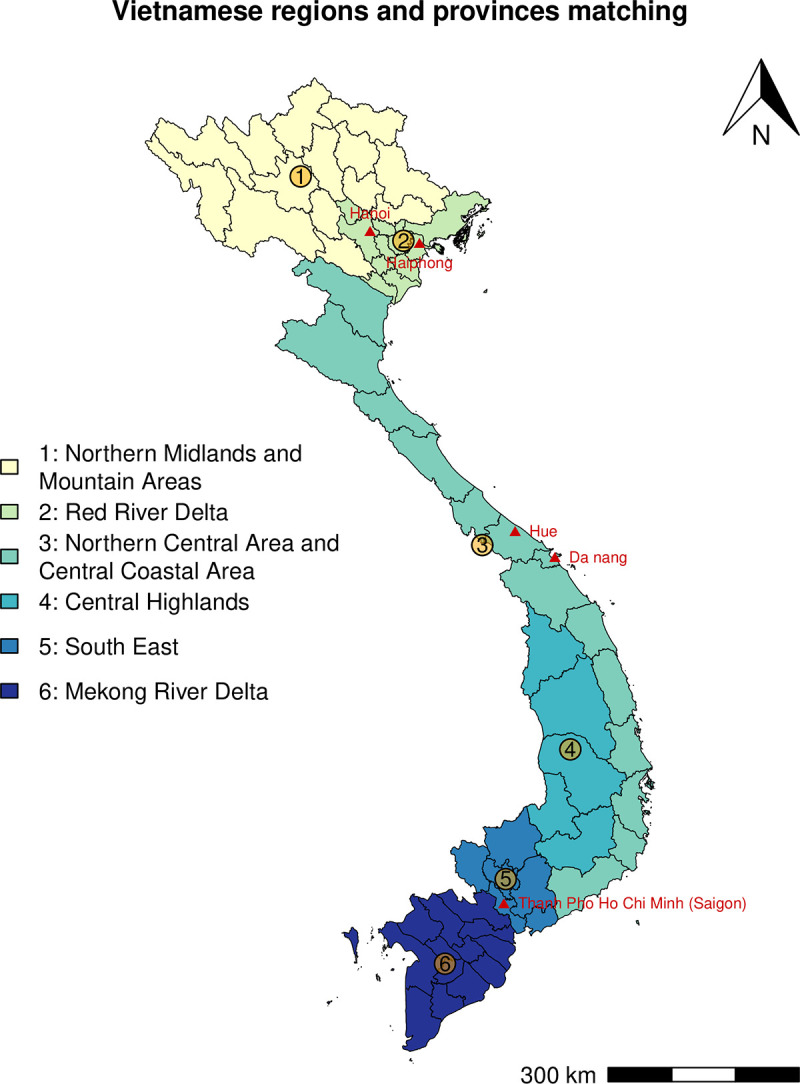
Vietnamese regions and provinces grouping. Regions are distinguished by colors. Province boundaries are shown. The major cities are highlighted in red. Map only shows mainland Vietnam.

Vietnam is divided into 63 provinces, which are grouped into six larger regions from north to south: the Northern Midlands and Mountain Areas (bordering China and Laos, with a significant ethnic minority population), the Red River Delta (with densely populated rural plains and its capital Hanoi), Northern Central and Central Coastal Areas (stretching from North to South Vietnam with Hue and Da Nang Cities), the Central Highlands (a mountainous region bordering Cambodia with ethnic minorities), the South East (a prosperous industrial region that includes Ho Chi Minh City), and the Mekong River Delta (a rich agricultural region at the southernmost tip of the country). Fig 1 illustrates the six Vietnam regions and provinces.

### Data

We compiled an extensive database for the SRB for the six regions of Vietnam (S1 Dataset). Table 1 summarizes the observations available by data source. There are 526 observations available across six Vietnamese regions. The reference years of these observations range from 1972 to 2019. In total, at least 2,933,093 birth records are included in the SRB database [the total number of births excludes those from Annual Population Change and Family Planning Survey (PCFPS) because they are unknown]. The details of the data processing steps are given in the S1 Appendix.

**Table 1:** Vietnamese regional SRB database.

Survey name	Number of SRB observations	Number of births (or households)
**1989 Census**	6	79,180§
**1997 DHS**	39	15,202
**1999 Census**	6	39,464§
**2002 DHS**	26	14,100
**2006 Annual PCFPS**	6	24,499†
**2007 Survey of Birth**	155	643,683
**2008 Survey of Birth**	6	7,645
**2008 Annual PCFPS**	6	23,597‡
**2009 Annual PCFPS**	6	–
**2009 Census**	6	262,272§
**2010 Annual PCFPS**	6	–
**2011 Annual PCFPS**	6	–
**2012 Annual PCFPS**	6	–
**2013 Annual PCFPS**	6	–
**2013–2014 MICS**	34	14,486
**2014 Intercensal Survey**	154	482,446
**2014 Annual PCFPS**	6	–
**2015 Annual PCFPS**	6	305,600 households¶
**2016 Annual PCFPS**	6	305,600 households¶
**2017 Annual PCFPS**	6	–
**2018 Annual PCFPS**	6	–
**2019 Annual PCFPS (Preliminary)**	6	–
**2019 Census**	6	1,374,615§
**total**	**526**	2,933,093*

DHS: Demographic and Health Survey. MICS: Multiple Indicator Cluster Survey. Annual PCFPS: Annual Population Change and Family Planning Survey (names of the survey may vary). Surveys are ranked by survey year. The number of births includes births within 25 years prior to the survey conducted.

†: from Table 1 of Guilmoto et al. [10].

‡: from page 5, “The analysis from the population change survey 2008” presentation.

§: population at age 0.

¶: the best information we have regarding the sample of these surveys.

*: the total number of births excludes those from Annual PCFPS because the individual-level data are unavailable.

Data sources include the decennial census rounds and surveys of different types. The 1997 and 2002 Demographic and Health Surveys (DHS), 2013–2014 Multiple Indicator Cluster Survey (MICS), and 2014 Intercensal Survey are nationally representative retrospective surveys collecting full birth histories from women aged 15–49 years. For these surveys, we only include births within 25 years prior to the year in which the surveys were conducted.

The PCFPS has been conducted almost annually by the Vietnamese General Statistical Office (GSO) since 2000 [26]. It records births from the previous 12 months from the date of the survey interview. The regional SRB data are only publicly available from 2006 onward for Annual PCFPS. In 2007 and 2008, the GSO conducted specific Surveys of Birth among health facilities across the country. The 2007 round included full birth histories, and the 2008 round only recorded information on the most recent births. Hence, we only use births in 2008 to approximate the SRB from the 2008 Survey of Birth (the sex ratio of births during earlier years is likely biased due to the stopping rules favoring male births).

Census data for 1989, 1999, and 2009 are available from the IPUMS-International [27] and from official tabulations for 2019 [28]. We use the population classified by age and sex from the census to compute the SRB by using the population at age zero and survival ratio from birth to age zero by sex.

To maintain the geographic consistency of the regional division used here, we group individual-level birth histories into regions according to the province for observations from DHS, MICS, and censuses. Table 1 in S1 Appendix lists the current 63 provinces and their corresponding regions.

### Bayesian hierarchical model for SRB

In the absence of published birth registration figures that would allow for the direct computation of the SRB, uncertainty is inherent in the estimation and forecasting of SRB trends because observations are derived from various data sources (censuses and sample surveys of different formats). It is crucial to be able to assess this uncertainty for policy decision-making. The Bayesian framework deployed here tackles this problem by providing a way to incorporate all measurements for Vietnamese regions while providing formal statistical credible intervals.

We developed a Bayesian hierarchical time series model to estimate the levels and trends in the SRB across six Vietnamese regions and produce probabilistic projections. The model for subnational SRB estimates and projections builds on the statistical model that is used to estimate and project SRB for all countries over time [5] but includes several modifications. The full model description is available in the S1 Appendix. We summarize the main approach and highlight the modifications from the existing model.

We model the SRB from a Vietnamese region as the sum of two parts: (i) normal level and (ii) SRB imbalance. For Part (i), the normal level is the product of the national baseline level of SRB fixed at 1.063 (available from [2]) and a region-year-specific factor that captures the natural year-by-year fluctuation within each Vietnamese region. The within-region time series is modeled with an autoregressive causal AR(1) model. Part (ii), the SRB imbalance, is assumed to be the product of a SRB imbalance detector and a region-year-specific sex ratio transition process (i.e., the rise, stabilization, and decline of the SRB).

The imbalance detector is assumed to be binary and takes value 1 if SRB imbalance exists and 0 otherwise. The detector follows a Bernoulli distribution with a region-specific probability of having SRB imbalance. The sex ratio transition is modeled with a trapezoid function and assumed nonnegative to approximate the effect of sex-selective abortion on SRB masculinity. In the trapezoid function, we estimate the following terms on the regional level: the starting year of the transition process, period length of the increase, plateau, the decline of the imbalance, and the maximum level of the imbalance. For all the region-specific parameters in Part (ii) regarding the SRB imbalance, they are modeled with hierarchical distributions with global mean and variance terms so that the information can be shared across regions; meanwhile, the differences across regions can be maintained if evaluated on the basis of data. The global mean and variance terms of the imbalance-related parameters are the model estimates of national-level imbalance in Vietnam [5].

In this study, we consider a Vietnamese region to be undergoing a sex ratio transition if the estimated probability of having SRB inflation in the region is at least 95%. We verified the AR(1) time series model structure with autocorrelation function plots (see S1 Appendix). We also conducted out-of-sample validation exercises and simulation analyses to check the prediction power of the model (see S1 Appendix). The validation results suggest that the model is reasonably calibrated and has good prediction performance.

## Results

The compiled database, annual estimates from 1980 to 2020 and projections from 2021 to 2050 for SRB by Vietnam region are available in S1–S3 Datasets respectively.

### Levels and trends before 2018

**Fig 2:**
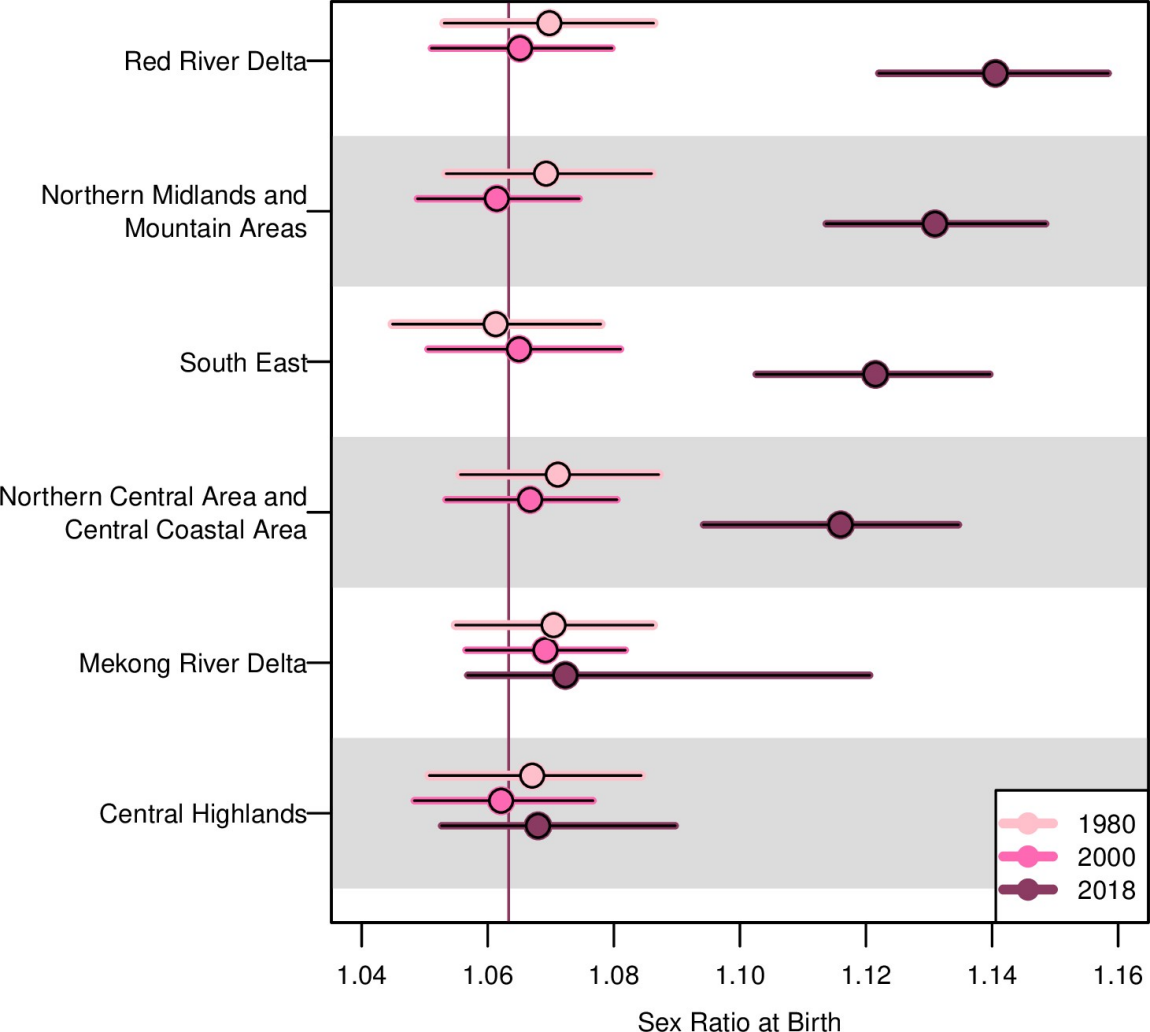
SRB estimates by Vietnam regions in 1980, 2000, and 2018. Median estimates are in dots. 95% credible intervals are in horizontal bars. The SRB national baseline for Vietnam as a whole is indicated by the vertical line at 1.063. The country’s regions are shown in descending order of the 2018 median estimates.

Fig 2 illustrates the estimated SRB among the six Vietnamese regions in the years 1980, 2000, and 2018. From 1980 to 2000, the SRB in Vietnam remains around the national SRB baseline 1.063 across all the six regions. Beginning in 2000, however, the between-region SRB variations started to increase, and by 2018, we estimate the SRB in four out of the six regions to be significantly higher than Vietnam’s natural level: in the Red River Delta, 1.141 with a 95% credible interval (1.122, 1.158); in the Northern Midlands and Mountain Areas, 1.131 (1.114, 1.148); in the South East, 1.122 (1.103, 1.140); and in the Northern Central and Central Coastal Areas, 1.116 (1.094, 1.135).

### SRB imbalances in Vietnam by region

Table 2 summarizes the model results of the analysis of SRB imbalances over time. We provide estimates of the schedule and intensity of these imbalances among the regions identified by our modeling as having skewed SRB levels. Four out of the six regions are identified as having an existing sex imbalance at birth: Northern Midlands and Mountain Areas, Northern Central and Central Coastal Areas, Red River Delta, and South East. For the Northern Midlands and Mountain Areas, Red River Delta, and South East, the model estimates a 100% probability of having SRB inflation. Meanwhile, it also suggests that the probability of a sex ratio transition is 98.2% for the Northern Central and Central Coastal Areas. The start year of SRB inflation is estimated at around 2001 for the four regions with a 95% credible interval around 4 to 6 years’ range. The corresponding SRB before the beginning of the sex ratio transition hardly varies, ranging from 1.063 (1.050, 1.076) in the Northern Midlands and Mountain Areas to 1.067 (1.053, 1.081) in the Northern Central and Central Coastal Areas.

**Table 2:** SRB imbalance in Vietnam by region.

Vietnamese region	Inflation probability	Start year	SRB at start year	Peak year	SRB at peak year
**Northern Central and Central Coastal Areas**	98.2%	2001 (1999, 2004)	1.067 (1.053, 1.081)	2015	1.117 (1.097, 1.135)
**Northern Midlands and Mountain Areas**	100%	2001 (1999, 2004)	1.063 (1.050, 1.076)	2016	1.132 (1.115, 1.149)
**Red River Delta**	100%	2001 (1999, 2002)	1.065 (1.051, 1.080)	2013	1.146 (1.129, 1.163)
**South East**	100%	2001 (1998, 2004)	1.066 (1.051, 1.084)	2013	1.122 (1.105, 1.139)

Posterior median estimates are numbers before the brackets. 95% credible intervals are the numbers inside the brackets.

The years with the maximum SRB are more spread out across Vietnamese regions compared to the start years. The SRB reached its maximum first in 2013 for the Red River Delta at 1.146 (1.129, 1.163) and the South East at 1.122 (1.105, 1.139), followed by the Northern Central and Central Coastal Areas at 1.117 (1.097, 1.135) in 2015, and finally, by the Northern Midlands and Mountain Areas at 1.132 (1.115, 1.149) in 2016.

### SRB projections for regions with current imbalances

**Fig 3:**
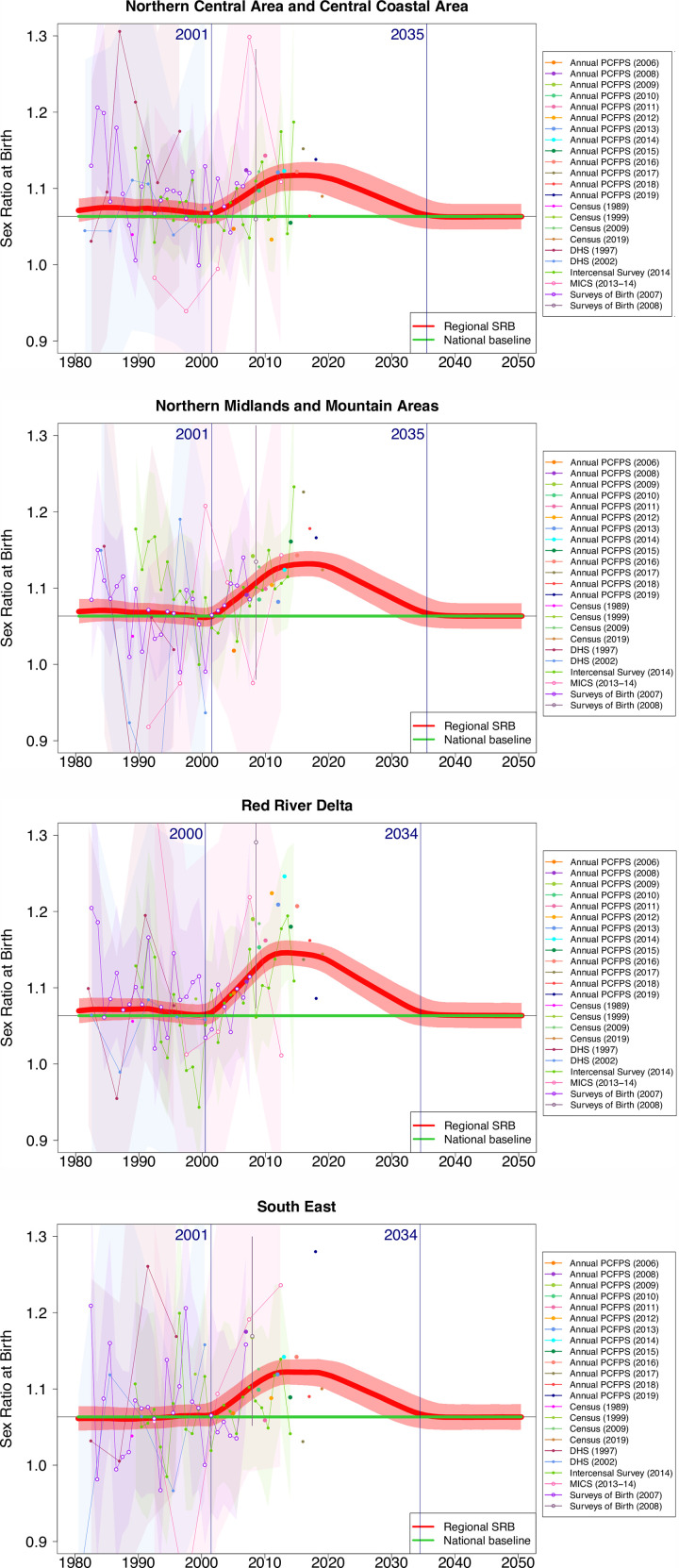
SRB estimates and projections for Vietnamese regions with imbalanced SRB during 1980–2050. The red line and shades are the median and 95% credible intervals of the region-specific SRB, respectively. The green horizontal line refers to the SRB baseline for Vietnam as a whole at 1.063 [2]. SRB observations are displayed with dots, and observations are connected with lines when obtained from the same source. Shaded areas around observation series represent the sampling variability in the series (quantified by two times the sampling standard errors). The start and end years of the sex ratio transition are shown by blue vertical lines.

Fig 3 provides a more detailed picture of the sex ratio transition in the four regions with skewed SRB levels; it combines observed SRB from the various data series used in this study with annual modeled estimates and uncertainty intervals. It may be noticed from Fig 3 that individual SRB observations vary from 0.9 to 1.3, an illustration of the volatility of regional estimates based on small birth samples.

The model highlights the stabilization of the SRB during the 2010s in all four affected regions; it also predicts a turnaround around 2020, with a gradual decline of the SRB back to normal levels during the next 15 years. The model projects that the SRB sex ratio transition process will end during the mid-2030s in the four regions of interest, starting with the Red River Delta and the South East in 2034.

**Fig 4:**
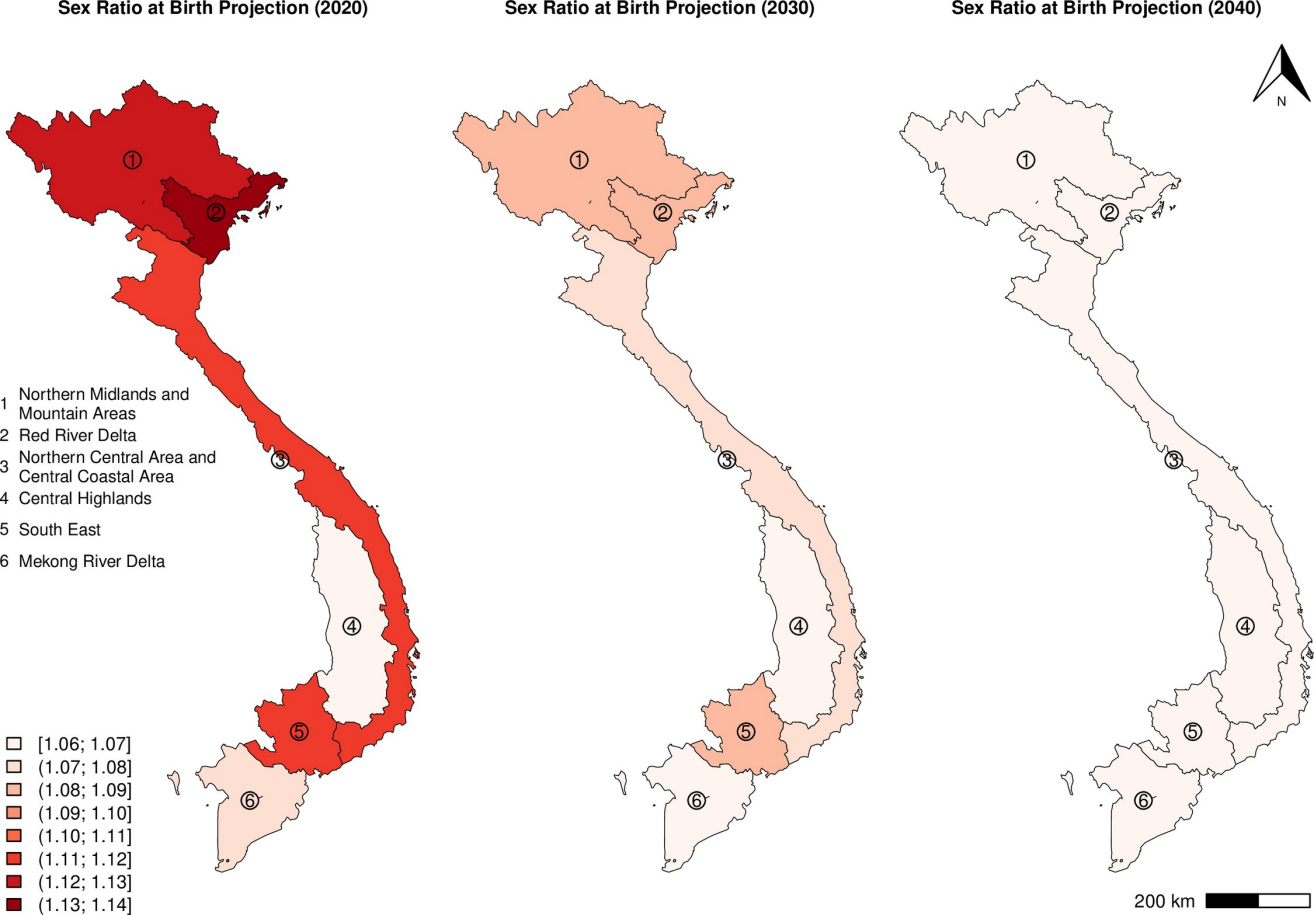
SRB median projections in Vietnam by region in 2020, 2030, and 2040. Maps only show mainland Vietnam.

Fig 4 provides an overview of the geographical disparities in SRB projection in 2020, 2030, and 2040. In general, the northern part of Vietnam is projected to have the highest SRB among all regions. The Red River Delta records the highest SRB level in 2020, but a subsequent decline in the SRB is parallel in all four regions; the Mekong River Delta and Central Highlands show no significant departure from the natural SRB level.

## Discussion and conclusion

This is the first study of SRB trends and projection in Vietnam using a subnational Bayesian approach. The model used is based on an extensive database compiled at a regional scale that includes all currently available survey and census sources. Not only does the model describe the trends in Vietnamese regions over the previous decades, but it also offers a unique disaggregated projection of the future SRB trends till 2050.

Vietnam experienced a rather late rise in its SRB despite the presence of entrenched son preference in most of the country and a rapid fertility decline observed since the 1980s, with fertility reaching replacement level at the end of the 20th century. According to our model results, the SRB started rising only in 2001 across regions; this date is slightly earlier than the result of a previous research based on births classified by month that dated the start of the rise to August 2003 in the country as a whole [29].

Most importantly, our study also allows for a detailed regional analysis of the SRB dynamics and confirms the rather heterogeneous picture of sex imbalances at birth in Vietnam. As in China and India, the SRB tends to vary widely across regions, with the coexistence within the same country of areas with no sex selection and areas exhibiting some of the highest levels observed in the world (e.g., 1.22 in Hunan Province of China in 2015 [30] and 1.18 in Haryana in India in 2016 [31]). These disparities are partly influenced by differences in the schedule of the fertility transition or by the unequal spread of modern health infrastructures. However, they are first accounted for by sizable regional variations in the demand for sex selection, namely, the intensity of the preference for a male child in local families. The results presented here show in particular that two regions of South Vietnam remained largely unaffected by the spread of prenatal sex selection observed in the rest of the country. The modeled SRB never significantly departed from the natural SRB level in these regions. This regional peculiarity can be attributed to the less acute need for a male child felt by couples in South Vietnam; this is due to the long-term influence of Khmer culture and the more egalitarian (bilateral) kinship system prevailing in this part of the country, rather distinct from the strict patrilineal family system prevailing in the North [12, 32].

In the four remaining regions (Northern Central and Central Coastal Areas, Northern Midlands and Mountain Area, Red River Delta, and South East), the SRB experienced a sustained rise over a duration of 12–15 years before reaching a peak in 2013–2016. Compared to other regions, the ascent of the SRB in the Red River Delta was notably faster, as it took only 12 years, and more pronounced, with the SRB culminating at 1.146 (1.129, 1.163) in 2013. The SRB recorded a more gradual progression in the three other regions, at an annual pace (measured as SRB increase per year), that is almost twice as slow as in the Red River Delta.

Another implication of the study findings is that the Red River Delta and the South East were the first regions to record a turnaround of the SRB, which started to slowly decline after 2013. The early decline of birth masculinity in these two regions is corroborated by the sex ratio by age derived from the 2019 census tabulations [14]. This decline affects all four regions in 2020 and is expected to accelerate over the next 15 years according to the projection model. Our model also posits that the SRB will first return to normalcy in the Red River Delta and South East (by 2034). At the end of 2030s, sex imbalances at birth should have completely vanished from all regions in Vietnam. While this study does not examine the factors behind the rise and fall of the SRB in Vietnamese regions, it can be assumed that the emergence of sex-selective abortions caused by the perceived need for a male offspring among Vietnamese couples has gradually receded as a consequence of both government policies and campaigns against sex selection introduced during the 2000s and under the indirect impact of the overall reduction in gender bias due to the rapid modernization of Vietnam’s economy and society. The changes in fertility or in access to sex-selective technology may have played a lesser role in determining the currently observed SRB downturn.

We may also stress that these scenarios are projections that cannot factor in the impact of the Vietnamese government’s current mobilization against gender bias. Several decrees and decisions have indeed been initiated to combat sex selection, starting with the ban of sex-selective abortions featuring in the 2003 Population Ordinance. More laws and initiatives have been introduced to reduce sex imbalances after the public recognition of skewed SRB levels at the time of the 2009 census [16]. The impact of these policy responses on gender attitudes of younger parents may further quicken the future decline of the SRB and the disappearance of sex imbalances at birth may occur several years before the projected date. The main lesson of these projections is precisely that local and national authorities need to focus their energy on the known determinants of sex imbalances at birth, most notably the prevalence of son preference and its impact on the couples’ fertility strategies, to accelerate attitudinal and behavioral change among young couples. The number of sex-selective abortions avoided in the coming years will automatically lower the size of the forthcoming surplus of adult males in the next thirty years.

The SRB model results of this study have a few limitations. First, we were not able to include external covariates such as the total fertility rate (TFR) in the model to assist the estimation and projection of the sex ratio transition. Although regional fertility data have been available annually since 2005, many regions have already reached a fertility level that is below 2.1, which is the fertility level in the year that the national SRB inflation is estimated to start [5]. For instance, in 2005, the TFR was 2.0 in the Mekong River Delta, 1.85 in the South East, and 2.06 in the Red River Delta. Hence, we made use of the national relation between the TFR and sex ratio transition to inform the start date of subnational SRB inflation. Furthermore, other potential covariates such as the usage of ultrasound technology for prenatal sex determination and the accessibility of abortion are in general not available for Vietnamese regions over time. Due to the lack of quality subnational data and covariates related to sex-selective abortion, our model does not detect great variety in the start year of occurrences of birth masculinity across regions. For instance, the Red River Delta, where there is evidence of staunch gender bias in favor of male children and where the Chinese historical influence has been the strongest, had long been assumed to be the region most prone to sex-selective behavior with a potential earlier rise in SRB compared to the rest of the country [33, 34]. Second, we had to impute the sampling errors for data from Annual PCFPS because the information on sampling design for these data sources is not available for such calculation. If such information is made available in the future, the model estimation and projection results could be slightly updated. Furthermore, if more reliable SRB data will be available in the future, the model-based and data-driven estimates and projections will be updated [35].

Our study highlights the importance of assessing Vietnam’s SRB levels and trends at the subnational level due to the ongoing change in sex ratio at the national level and the great heterogeneity in culture and demography across geographic locations. The study findings confirm the distinct schedule of the regional sex ratio transition in the country but show that the SRB should have returned to normalcy in all regions within 15 years under the model assumptions. Information related to sex-selective abortion at the subnational level should form the basis of targeted interventions to accelerate the decline of prenatal sex selection and alleviate the burden of missing female births and future sex imbalances in the adult population of Vietnam.

## Supporting information

S1 Appendix Data preprocessing, model specifications, computing details, validation results, and additional figures and tables. Available at: https://doi.org/10.6084/m9.figshare.14152979.
(PDF)

S1 Dataset Sex ratio at birth by Vietnam region database. Available at: https://doi.org/10.6084/m9.figshare.14724636.
(XLSX)

S2 Dataset Sex ratio at birth estimates by Vietnam region from 1980 to 2020. Available at: https://doi.org/10.6084/m9.figshare.14724678.
(XLSX)

S3 Dataset Sex ratio at birth projections by Vietnam region from 2021 to 2050. Available at: https://doi.org/10.6084/m9.figshare.14724696.
(XLSX)
