# Supplementary material for: Sex ratio at birth in Vietnam among six subnational regions during 1980–2050, estimation and probabilistic projection using a Bayesian hierarchical time series model with 2.9 million birth records
Source: PLoS One. 2021 Jul 14;16(7):e0253721. doi: 10.1371/journal.pone.0253721 (PMC8279334; doi:10.1371/journal.pone.0253721)
Supplement: S1 Appendix — Available at: https://doi.org/10.6084/m9.figshare.14152979. (PDF) [file pone.0253721.s001.pdf]

S1 Appendix
Sex ratio at birth in Vietnam among six subnational regions during 1980–2050, estimation and probabilistic projection using a Bayesian hierarchical time series model with 2.9 million birth records

Fengqing Chao*[1], Christophe Z. Guilmoto[2], and Hernando Ombao[1]

[1]Statistics Program, Computer, Electrical and Mathematical Sciences and Engineering Division, King Abdullah University of Science and Technology (KAUST), Thuwal, Saudi Arabia

[2]CEPED/IRD, Centre de Sciences Humaines, New Delhi, India

June 15, 2021

*Corresponding author (FC); Email: fengqing.chao@kaust.edu.sa

Contents

1 Data Pre-processing **1**
1.1 Matching Vietnam provinces to region . . . 1
1.2 Compute SRB from censuses . . . 1
1.3 Sampling errors for data based on full birth histories . . . 2
1.4 Stochastic errors for data based on censuses . . . 2
1.5 Imputing sampling errors for Annual PCFPS data . . . 3

2 Model Summary **3**
2.1 Notations . . . 3
2.2 Model for Sex Ratio at Birth by Vietnam region . . . 3
2.3 Data quality model . . . 5
2.4 Priors . . . 5
2.5 Statistical Computing . . . 6

3 Model Validation **6**
3.1 Out-of-Sample Validation . . . 6
3.2 One-Region Simulation . . . 7

4 Validation and Simulation Results **7**

List of Tables

1 Vietnam regions and provinces matching . . . 1
2 Notation summary . . . 4
3 Validation and simulation results for left-out SRB observations . . . 9
4 Validation results for estimates based on training set . . . 9

List of Figures

1 Autocorrelation Function (ACF) for AR(1) model results, by Vietnam region . . . 8

List of Abbreviations

ACF	Autocorrelation Function
AR(1)	Auto-Regressive model of order 1
CV	coefficient of variation
DHS	Demographic and Health Survey
GSO	General Statistics Office
MCMC	Markov chain Monte Carlo
MICS	Multiple Indicator Cluster Survey
PCFPS	Population Change and Family Planning Survey
SRB	Sex Ratio at Birth
TFR	Total Fertility Rate

1 Data Pre-processing

1.1 Matching Vietnam provinces to region

We use the Vietnam region names from the General Statistics Office (GSO) of Vietnam. Table 1 lists the Vietnam regions of interest (Vietnam GSO region) and corresponding region names in DHS microdata files.

**Vietnam GSO region**	**Vietnam DHS region**	**[63] Province**
**Red River Delta**	Red River Delta	[11] Hà Nội; Vĩnh Phúc; Bắc Ninh; Quảng Ninh; Hải Dương; Hải Phòng; Hưng Yên; Thái Bình; Hà Nam; Nam Định; Ninh Bình
**Northern midlands and mountain areas**	Northern Uplands	[14] Hà Giang; Cao Bằng; Bắc Kạn; Tuyên Quang; Lào Cai; Yên Bái; Thái Nguyên; Lạng Sơn; Bắc Giang; Phú Thọ; Điện Biên; Lai Châu; Sơn La; Hoà Bình
**Northern Central area and Central coastal area**	North Central; Central Coast	[14] Thanh Hóa; Nghệ An; Hà Tĩnh; Quảng Bình; Quảng Trị; Thừa Thiên Huế; Đà Nẵng; Quảng Nam; Quảng Ngãi; Bình Định; Phú Yên; Khánh Hòa; Ninh Thuận; Bình Thuận
**Central Highlands**	Central Highlands	[5] Đắk Lắk; Lâm Đồng; Đắk Nông; Gia Lai; Kon Tum
**South East**	South East	[6] Bình Phước; Tây Ninh; Bình Dương; Đồng Nai; Bà Rịa - Vũng Tàu; Hồ Chí Minh
**Mekong River Delta**	Mekong River Delta	[13] Long An; Tiền Giang; Bến Tre; Trà Vinh; Vĩnh Long; Đồng Tháp; An Giang; Kiên Giang; Cần Thơ; Hậu Giang; Sóc Trăng; Cà Mau; Bạc Liêu

Table 1: **Vietnam regions and provinces matching.** The red numbers at the beginning of each cell refers to the number of provinces belong to each region. Hà Tây belongs to Red River Delta (it is not shown in the table because it merged into Hà Nội province).

1.2 Compute SRB from censuses

We use the population at age zero from census to compute the sex ratio of population at age zero. Then we compute the SRB using the sex ratio of population divided by the sex ratio of survival rate between age zero to one year old:

$$ {}_1SR_0 = \frac{{}_1N_0^M}{{}_1N_0^F}, \quad (1) $$

$$ {}_1s_0 = \frac{{}_1L_0^M}{{}_1L_0^F}, \quad (2) $$

$$ SRB = \frac{{}_1SR_0}{{}_1s_0}. \quad (3) $$

${}_1N_0^M$ and ${}_1N_0^F$ are the number of male and female population below age one year old respectively. ${}_1L_0^M$ and ${}_1L_0^F$ are the number of years lived between age zero and one for male and female respectively.

1.3 Sampling errors for data based on full birth histories

DHS, MICS, 2007 Survey of Birth and 2014 Intercensal Survey provide individual-level data with the full birth history for each woman at reproductive age interviewed during the survey fieldwork period. We calculate the sampling error for log-transformed SRB for DHS data series using the jackknife method [1, 2, 3]. For a certain data series with full birth histories, let U denote the total number of clusters. The uth partial prediction of SRB is given by:

$$y_{-u} = \frac{\sum_{n=1}^{N} \mathbb{I}_n(x_n = \text{male}; d_n \neq u) w_n}{\sum_{n=1}^{N} \mathbb{I}_n(x_n = \text{female}; d_n \neq u) w_n}, \text{ for } u \in \{1, \ldots, U\},$$

where n indexes the live births in each state-survey-year, N is the total number of live births. x_n is the sex for the nth live birth. d_n is the cluster number for the nth live birth. w_n is the sampling weight for the nth live birth. $\mathbb{I}_n(\cdot) = 1$ if the condition inside brackets is true and $\mathbb{I}_n(\cdot) = 0$ otherwise. The uth pseudo-value estimate of the SRB on log-scale is:

$$\log(y)_u^* = U \log(y') - (U-1)\log(y_{-u}), \text{ where}$$

$$y' = \frac{\sum_{n=1}^{N} \mathbb{I}_n(x_n = \text{male}) w_n}{\sum_{n=1}^{N} \mathbb{I}_n(x_n = \text{female}) w_n}.$$

The sampling variance is:

$$\sigma^2 = \frac{\sum_{u=1}^{U} (\log(y)_u^* - \overline{\log(y)_u^*})^2}{U(U-1)}, \text{ where}$$

$$\overline{\log(y)_u^*} = \frac{1}{U} \sum_{u=1}^{U} \log(y)_u^*.$$

The annual log-transformed SRB observations are merged such that the coefficient of variation (CV) for log-transformed SRB is below 0.1 or the merged period reached 5 years [4]. For a certain data series, let $\{t_n, t_{n-1}, \cdots, t_1\}$ be the years with recorded births from recent to past. The merge starts from the most recent year t_n:

Merging process for data based on full birth histories

1: **for** $t \in \{t_n, t_{n-1}, \cdots, t_1\}$ **do**
2: **if** $t = t_n$ **then**
3: Compute σ as explained in above. Compute CV= $\sigma / \log(y)_u^*$
4: **if** CV < 0.1 **or** $t_n - t_{n-1} > 1$ **or** $t_{n+1} - t_n = 5$ **then**
5: **stop** and move to the previous time point
6: **else**
7: Repeat steps 3–5 based on births from t_n and t_{n-1}

The above procedures of computing sampling error and merging observation periods are performed for each data series with full birth history information.

1.4 Stochastic errors for data based on censuses

We assume that the census coverage of population is close to 100%. Hence, the main uncertainty from the census data are stochastic errors. We used a Monte Carlo simulation to approximate the stochastic variance. For a region-year, the gth simulated number of male live births $B^{M(g)}$ is obtained as:

$$B^{M(g)} \sim \mathcal{B}(B_{\text{rep}}, p_{\text{rep}}^M), \text{ for } g \in \{1, \ldots, G\},$$

where $\mathcal{B}$ denotes a binomial distribution, G is the total number of simulations, B_{rep} is the total number of live births as reported in VR data, and p_{rep}^{M} is the reported proportion of male live births among the reported total live births. The corresponding gth simulation for SRB is given by:

$$y^{(g)} = \frac{B^{M(g)}}{B_{\text{rep}} - B^{M(g)}}, \text{ for } g \in \{1, \ldots, G\}.$$

The stochastic error for SRB on log-scale is:

$$\sigma_{\text{ST}} = \sqrt{\frac{1}{G-1}\sum_{g=1}^{G}\left(\log(y^{(g)}) - \overline{\log(y)}\right)^2}, \text{ where } \overline{\log(y)} = \frac{1}{G}\sum_{g=1}^{G}\log(y^{(g)}).$$

1.5 Imputing sampling errors for Annual PCFPS data

Given that the individual-level birth records from Annual PCFPS are not publicly available, we are not able to directly compute the sampling errors associated with Annual PCFPS data. Hence, we imputed the sampling error for observed SRB from Annual PCFPS at 0.05, which is the median sampling error from 2007 and 2008 Survey of Birth and 2014 Intercensal Survey.

2 Model Summary

2.1 Notations

Table 2 summarizes the notations and indexes used in this paper. $\mathcal{N}(\mu, \sigma^2)$ refers to a normal distribution with mean μ and variance σ^2. $t_3(\mu, \sigma^2)$ refers to a Student-*t* distribution with degree of freedom 3, mean μ and variance σ^2. $\mathcal{U}(a, b)$ denotes a continuous uniform distribution with lower and upper bounds at a and b respectively.

2.2 Model for Sex Ratio at Birth by Vietnam region

The model is largely based on the model described in [7]. In this study, we made a few modifications in the model to better address the data quality and availability of provincial SRB in Vietnam. The outcome of interest $\Theta_{r,t}$, the SRB in Vietnam region r in year t is modeled as:

$$\begin{aligned}
\Theta_{r,t} &= b\Phi_{r,t} + \delta_r \alpha_{r,t}, \\
\log(\Phi_{r,t}) &\sim \mathcal{N}(0, (1-\rho^2)/\sigma_\epsilon^2), \text{if } t = 1980, \\
\log(\Phi_{r,t}) &= \rho \log(\Phi_{r,t-1}) + \epsilon_{r,t}, \text{if } t \in \{1981, \cdots, 2050\}, \\
\epsilon_{r,t} &\overset{\text{i.i.d.}}{\sim} \mathcal{N}(0, \sigma_\epsilon^2).
\end{aligned}$$

$b = 1.063$ is the SRB baseline level for the entire Vietnam. The Vietnam SRB baseline b is estimated based on national SRB observations from Vietnam before reference year 1970 [5, 6]. $\Phi_{r,t}$ follows an AR(1) times series model on the log scale to capture the natural fluctuations of SRB within each region over time. $\rho = 0.9$ and $\sigma_\epsilon = 0.004$ based on previous study [5, 6]. Instead of estimating ρ and σ_ϵ, we use the estimated values from prior study [5, 6] since the study is based on an extensive national SRB database and the estimates of the parameters are robust.

δ_r is the binary identifier of the sex ratio transition, following a Bernoulli distribution:

$$\begin{aligned}
\delta_r | \pi_r &\sim \mathcal{B}(\pi_r), \text{ for } r \in \{1, \cdots, 6\}, \\
\text{logit}(\pi_r) | \mu_\pi, \sigma_\pi &\sim \mathcal{N}(\mu_\pi, \sigma_\pi^2), \text{ for } r \in \{1, \cdots, 6\}.
\end{aligned}$$

Symbol	Description
	Indexes
i	Indicator for the ith SRB observation across all region-years, $i \in \{1, \ldots, 526\}$.
t	Indicator for year, $t \in \{1980, \ldots, 2050\}$.
r	Indicator for Vietnam region, $r \in \{1, \ldots, 6\}$.
	Unknown Parameters
$\Theta_{r,t}$	The model fitting for the true SRB for Vietnam region r in year t.
$\Phi_{r,t}$	The region-year-specific multiplier for capturing the natural fluctuation in SRB around the national baseline b for Vietnam region r in year t.
$\alpha_{r,t}$	The SRB imbalance for Vietnam region r in year t.
$t_{0,r}$	The start year of the SRB inflation for Vietnam region r.
δ_r	The indicator of the presence ($\delta_r = 1$) or absence ($\delta_r = 0$) of SRB inflation in Vietnam region r.
ξ_r	The maximum level of the SRB inflation for Vietnam region r.
$\lambda_{1,r}$	The period length for the stage of increase of the sex ratio transition for Vietnam region r.
$\lambda_{2,r}$	The period length for the stage of stagnation of the sex ratio transition for Vietnam region r.
$\lambda_{3,r}$	The period length for the stage of decrease back to national SRB baseline of the sex ratio transition for Vietnam region r.
ω	The non-sampling error for all the observations.
	Known Quantities
y_i	The ith SRB observation.
σ_i	The sampling error for the ith SRB observation as computed in Section 1.3.
b	The baseline level of SRB for the whole Vietnam [5], where $b = 1.063$.
ρ	The autoregressive indicator for $\Phi_{r,t}$, where $\rho = 0.9$ [5, 6].
σ_ϵ	The SD of distortion parameter for $\Phi_{r,t}$, where $\sigma_\epsilon = 0.004$ [5, 6].

Table 2: **Notation summary.**

The logit transformation ensures that the probability parameter π_r lies in the interval [0, 1]. The logit-transformed π_r follows a hierarchical normal distribution with a global mean and variance μ_π and σ^2_π.

$\alpha_{r,t}$ refers to the region-specific SRB imbalance process, and is modeled by a trapezoid function to represent the increase, stagnation, and decrease of the transition stages:

$$\begin{aligned}
\alpha_{r,t} &= \begin{cases} (\xi_r/\lambda_{1,r})(t-\gamma_r), & t_{0,r} < t < t_{1,r} \\ \xi_r, & t_{1,r} < t < t_{2,r} \\ \xi_c - (\xi_c/\lambda_{3,r})(t - t_{2,r}), & t_{2,r} < t < t_{3,r} \\ 0, & t < t_{0,r} \text{ or } t > t_{3,r} \end{cases} \\
t_{1,r} &= t_{0,r} + \lambda_{1,r}, \\
t_{2,r} &= t_{1,r} + \lambda_{2,r}, \\
t_{3,r} &= t_{2,r} + \lambda_{3,r}, \\
t_{0,r}|\sigma_{t0} &\sim t_3(2001, \sigma^2_{t0}), \text{ for } r \in \{1, \cdots, 6\}.
\end{aligned}$$

The start year of SRB inflation $t_{0,r}$ incorporates the fertility squeeze effect by using the data of total fertility rate (TFR). $t_{0,r}$ is modeled with a Student-t distribution with degree of freedom 3 with mean at 2001 which indicates the year in which the TFR in Vietnam region r declines to 2.0 [7]. We use the year 2001 to determine the mean of the distribution for $t_{0,r}$ because this is the estimated year in which Vietnam may start the sex ratio transition [7]. The low degrees of freedom for the Student-t distribution is needed to capture higher uncertainty at the tails for the distribution of $t_{0,r}$. Please refer to [7] for more details on the model assumption for $\alpha_{r,t}$.

2.3 Data quality model

y_i is the ith observed SRB from region $r[i]$ in year $t[i]$, where i indexes all the SRB observations across regions over time. y_i is assumed to follow a normal distribution on the log scale with mean at $\log(\Theta_{r[i],t[i]})$ (explained as above) and variance at the sum of σ^2_i and ω^2:

$$\log(y_i)|\Theta_{r[i],t[i]}, \omega \sim \mathcal{N}(\log(\Theta_{r[i],t[i]}), \sigma^2_i + \omega^2), \text{ for } i \in \{1, \cdots, 526\},$$

σ^2_i is the sampling/stochastic error variance for $\log(y_i)$ which reflects the uncertainty associated with log-scaled SRB observations due to survey sampling design or underlying natural fluctuations. σ^2_i is known and are calculated as explained in Section 1.3 and Section 1.4. ω^2 represents the non-sampling error that can be minimized but cannot be entirely eradicated due to causes such as non-responses, data input errors.

Note that we use both r, t and i to index the observations instead of only using r and t. This is because for some region r year t, there are multiple data point available. Although the mean of each region r year t is the same at $\Theta_{r,t}$, the variances of these data points are different at $\sigma^2_i + \omega^2$.

2.4 Priors

Informative priors are assigned to region-level parameters related to the sex ratio transition: the maximum level of SRB inflation ξ_r, the period lengths for the stages of increase, stagnation and decrease as $\lambda_{1,r}$, $\lambda_{2,r}$ and $\lambda_{3,r}$ respectively. The means of the prior distributions are from the systematic study [7, 8] which modeled the sex ratio transition for multiple countries including Vietnam. The standard deviations of the prior distribution are set such that the coefficient of variations (CV; defined as the ratio between mean and standard deviation) are 0.1. The informative priors could assist in modeling the sex ratio transition on the region level in Vietnam by making use of the corresponding information on the national level. For

$r \in \{1, \cdots, 6\}$:

$$\begin{aligned} \xi_r &\sim \mathcal{N}(0.07, 0.007^2), \\ \lambda_{1,r} &\sim \mathcal{N}(10.1, 1.0^2), \\ \lambda_{2,r} &\sim \mathcal{N}(7.7, 0.8^2), \\ \lambda_{3,r} &\sim \mathcal{N}(16.1, 1.6^2). \end{aligned}$$

Vague priors are assigned to parameters related to the indicator for detecting the existence of a sex ratio transition, the SD of start year and the non-sampling error:

$$\begin{aligned} \text{inverse-logit}(\mu_\pi) &\sim \mathcal{U}(0, 1), \\ \sigma_\pi &\sim \mathcal{U}(0, 2), \\ \sigma_{t0} &\sim \mathcal{U}(0, 10), \\ \omega &\sim \mathcal{U}(0, 0.5). \end{aligned}$$

2.5 Statistical Computing

We obtained posterior samples of all the model parameters and hyper parameters using a Markov chain Monte Carlo (MCMC) algorithm, implemented in the open source softwares `R 3.6.1` [9] and `JAGS 4.3.0` [10], using `R`-packages **R2jags** [11] and **rjags** [12]. Results were obtained from 10 chains with a total number of 5,000 iterations in each chain, while the first 10,000 iterations were discarded as burn-in. After discarding burn-in iterations and proper thinning, the final posterior sample size for each parameter by combining all chains is 25,000. Convergence of the MCMC algorithm and the sufficiency of the number of samples obtained were checked through visual inspection of trace plots and convergence diagnostics of Gelman and Rubin [13], implemented in the **coda** `R`-package [14].

3 Model Validation

We assess the inflation model performance via two approaches: 1) out-of-sample validation; and 2) one-region simulation.

3.1 Out-of-Sample Validation

We leave out 37% of the data points since the data collection year 2014 instead of reference year, which has been used in assessing model performance for demographic indicators largely based on survey data [15, 16, 17, 18]. After leaving out data, we fit the model to the training data set, and obtain point estimates and credible intervals that would have been constructed based on the available data set in the survey year selected.

We calculate median errors and median absolute errors for the left-out observations, where errors are defined as: $e_j = y_j - \widetilde{y}_j$, where $\widetilde{y}_j$ refers to the posterior median of the predictive distribution based on the training data set for the jth left-out observation y_j. Coverage is given by $1/J \sum \mathbb{I}[y_j \geq l_j]\mathbb{I}[y_j \leq u_j]$, where J refers to the number of left-out observations, and l_j and u_j correspond to the lower and upper bounds of the 95% prediction interval for the jth left-out observation y_j. The validation measures are calculated for 1000 sets of left-out observations, where each set consists one randomly selected left-out observation from each Vietnam region. The reported validation results are based on the mean of the outcomes from the 1000 sets of left-out observations.

For the point estimates based on full data set and training data set, errors for the true level of SRB are defined as $e(\Theta)_{r,t} = \widehat{\Theta}_{r,t} - \widetilde{\Theta}_{r,t}$, where $\widehat{\Theta}_{r,t}$ is the posterior median for region r in year t based on the full

data set, and $\widetilde{\Theta}_{r,t}$ is the posterior median for the same region-year based on the training data set. Similarly, the error for the sex ratio transition process with probability is defined as $e(\alpha\delta)_{r,t} = \widehat{\alpha}_{r,t}\widehat{\delta}_r - \widetilde{\alpha}_{r,t}\widetilde{\delta}_r$. Coverage is computed in a similar manner as for the left-out observations, based on the lower and upper bounds of the 95% credible interval of $\widetilde{\Theta}_{r,t}$ from the training data set.

3.2 One-Region Simulation

We assess the inflation model performance by one-region simulation. For each of the six Vietnam regions, we consider all data points as test data and simulate the SRB using the posterior samples of the global parameters from the sex ratio transition model (obtained using the full dataset).

The gth simulated SRB $\Theta^{(g)}_{r,t}$ for region r in year t, and the gth simulated SRB $\Theta^{(g)}_{r[j],t[j]}$ for the jth left-out data point for region $r[j]$ in year $t[j]$ are obtained as follows for $g \in \{1, \ldots, G\}$: $\Theta^{(g)}_{r,t} = b\Phi^{(g)}_{r,t} + \alpha^{(g)}_{r,t}\delta^{(g)}_r$, where $\Phi^{(g)}_{r[j],t[j]}$, $\alpha^{(g)}_{r[j],t[j]}$ and $\delta^{(g)}_r$ are simulated to refer to a "new" region, without taking into account any region-specific data, following the model specification for these parameters. $\alpha_{r,t}$ and δ_r are simulated using the posterior samples of all parameters and hyper-parameters related to them. After generating the simulated values, we calculate the same set of results as described in Section 3.1 on out-of-sample validation.

4 Validation and Simulation Results

Figure 1 shows the model results of the Autocorrelation Function (ACF) for AR(1) structure. The ACF values shown in Figure 1 are calculated as:

$$\text{ACF}_h = \frac{\text{Cov}(\Phi_{r,t}, \Phi_{r,t-h})}{\text{Var}(\Phi_{r,t})}, \text{ for } r \in \{1, \cdots, 6\},$$

where $t = 2050$, and $h \in \{1, 2, \cdots, 70\}$. Given that the autoregressive indicator for $\Phi_{r,t}$ is set to positive ($\rho = 0.9$ [5, 6]), the ACF across Vietnam regions decreases exponentially to zero as the lag increases.

Table 3 summarizes the results related to the left-out SRB observations for the out-of-sample validation exercise and the one-country simulation. Median errors and median absolute errors are very close to zero for left-out observations. The coverage of 95% and 80% prediction intervals are symmetrical and more conservative than expected. The wider-than-expected prediction interval for left-out observations are mainly due to the greater uncertainty associated in more recent observations.

Table 4 shows results for the comparison between model estimates obtained based on the full dataset and based on the training set for the out-of-sample validation exercise. We look at the model estimates for the true SRB $\Theta_{r,t}$ and the inflation process with country-specific probability $\delta_r\alpha_{r,t}$. Median errors and the median absolute errors are close to zero.

In summary, the validation results indicate reasonably good calibrations and predicting power of the inflation model with conservative credible intervals.

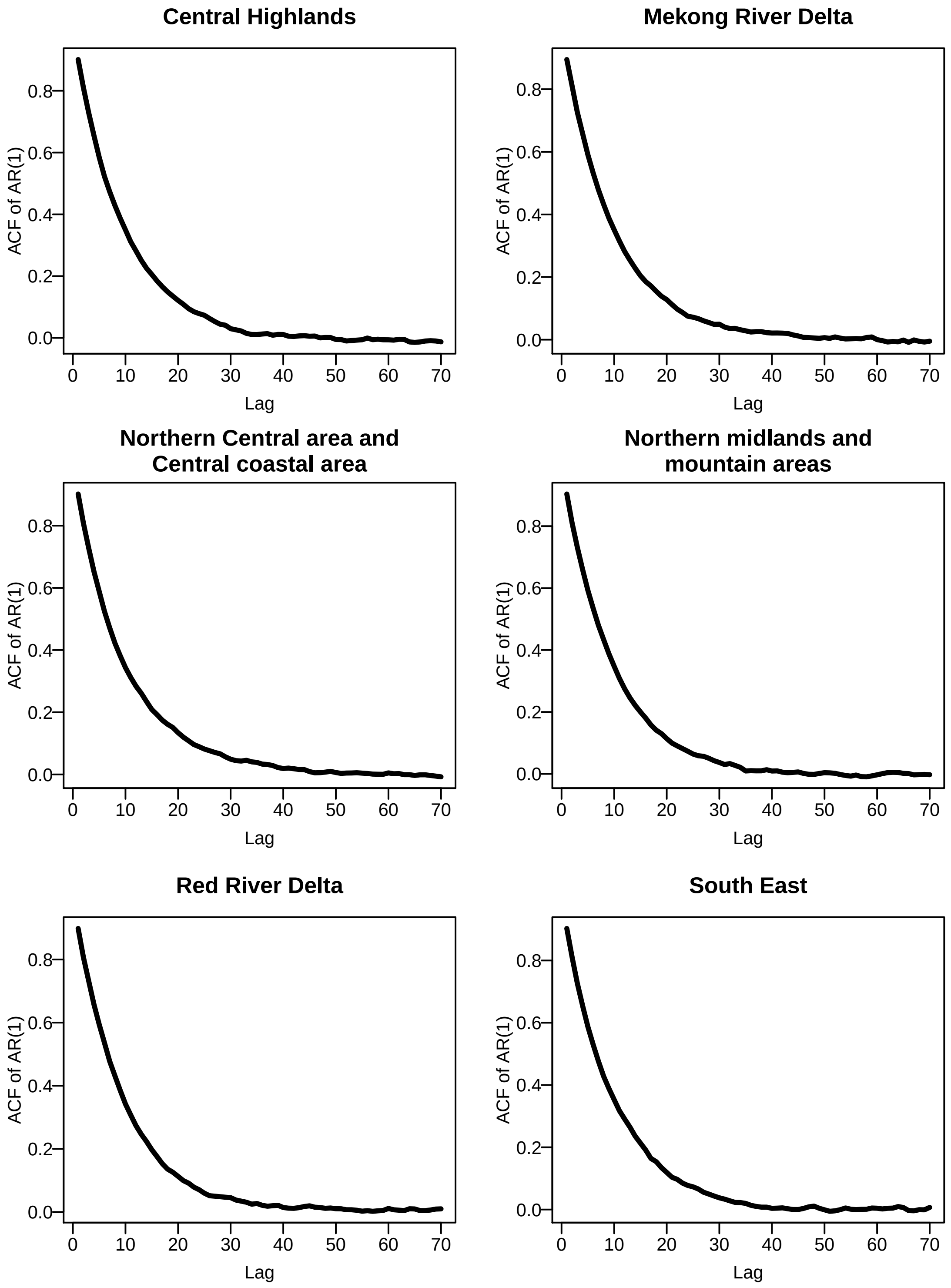

Figure 1: **Autocorrelation Function (ACF) for AR(1) model results, by Vietnam region.**

	Validation Out-of-Sample	Simulation
# region in test dataset	6	6
Median error	-0.002	0.009
Median absolute error	0.032	0.042
Below 95% prediction interval (%)	0.4	0.4
Above 95% prediction interval (%)	0.0	1.0
**Expected (%)**	**2.5**	**2.5**
Below 80% prediction interval (%)	1.9	3.4
Above 80% prediction interval (%)	2.6	7.4
**Expected (%)**	**10**	**10**

Table 3: **Validation and simulation results for left-out SRB observations.** Error is defined as the difference between a left-out SRB observation and the posterior median of its predictive distribution. SRB observations with data collection years since 2014 are left out. Numbers in the parentheses after the proportions indicate the average number of left-out observations fall below or above their respective 95% and 80% prediction intervals.

**Model Validation**	$\Theta_{r,t}$			$\delta_r\alpha_{r,t}$		
**(Out-of-Sample)**	**1995**	**2005**	**2013**	**1995**	**2005**	**2013**
Median error	0.000	0.006	0.002	0.000	-0.002	-0.001
Median absolute error	0.001	0.006	0.006	0.000	0.002	0.003
Below 95% credible interval (%)	0.0	0.0	0.0	0.0	0.0	0.0
Above 95% credible interval (%)	0.0	0.0	0.0	0.0	0.0	0.0
**Expected (%)**	**≤2.5**	**≤2.5**	**≤2.5**	**≤2.5**	**≤2.5**	**≤2.5**
Below 80% credible interval (%)	0.0	0.0	0.0	0.0	0.0	0.0
Above 80% credible interval (%)	0.0	0.0	0.0	0.0	0.0	0.0
**Expected (%)**	**≤10**	**≤10**	**≤10**	**≤10**	**≤10**	**≤10**

Table 4: **Validation results for estimates based on training set.** Error is defined as the differences between a model estimate (i.e. $\Theta_{r,t}$ or $\delta_r\alpha_{r,t}$) based on full dataset and training set. The proportions refer to the proportions (%) of countries in which the median estimates based on the full dataset fall below or above their respective 95% and 80% credible intervals based on the training set.

References

[1] Efron B, Gong G. A leisurely look at the bootstrap, the jackknife, and cross-validation. The American Statistician. 1983;37(1):36–48.

[2] Efron B, Tibshirani RJ. An introduction to the bootstrap. In: An introduction to the bootstrap. CHAPMAN & HALLI/CRC; 1994. .

[3] International ICF. Demographic and Health Survey Sampling and Household Listing Manual. Calverton, Maryland, U.S.A.: MEASURE DHS; 2012. p. 78–79. Available from: https://dhsprogram.com/pubs/pdf/DHSM4/DHS6_Sampling_Manual_Sept2012_DHSM4.pdf.

[4] Pedersen J, Liu J. Child mortality estimation: appropriate time periods for child mortality estimates from full birth histories. PLoS medicine. 2012;9(8).

[5] Chao F, Gerland P, Cook AR, Alkema L. Systematic assessment of the sex ratio at birth for all countries and estimation of national imbalances and regional reference levels. Proceedings of the National Academy of Sciences. 2019;116(19):9303–9311.

[6] Chao F, Gerland P, Cook AR, Alkema L. Web Appendix Systematic assessment of the sex ratio at birth for all countries and estimation of national imbalances and regional reference levels. 2019;DOI: 10.6084/m9.figshare.12442373. Available at https://www.pnas.org/content/pnas/suppl/2019/04/10/1812593116.DCSupplemental/pnas.1812593116.sapp.pdf.

[7] Chao F, Gerland P, Cook AR, Alkema L. Global estimation and scenario-based projections of sex ratio at birth and missing female births using a Bayesian hierarchical time series mixture model. arXiv preprint arXiv:200607101. 2020;.

[8] Chao F, Gerland P, Cook AR, Guilmoto CZ, Alkema L. Projecting sex imbalances at birth at global, regional, and national levels from 2018 to 2100: scenario-based Bayesian probabilistic projections of the sex ratio at birth and missing female births. SocArXiv. 2021;DOI: 10.31235/osf.io/zps2m. Available at https://osf.io/preprints/socarxiv/zps2m/.

[9] R Core Team. R: A Language and Environment for Statistical Computing. Vienna, Austria; 2019. Available from: https://www.R-project.org/.

[10] Plummer M, et al. JAGS: A program for analysis of Bayesian graphical models using Gibbs sampling. In: Proceedings of the 3rd international workshop on distributed statistical computing. vol. 124. Vienna, Austria.; 2003. p. 10.

[11] Su YS, Yajima M. R2jags: Using R to Run 'JAGS'; 2015. R package version 0.5-7. Available from: https://CRAN.R-project.org/package=R2jags.

[12] Plummer M. rjags: Bayesian Graphical Models using MCMC; 2018. R package version 4-8. Available from: https://CRAN.R-project.org/package=rjags.

[13] Gelman A, Rubin DB, et al. Inference from iterative simulation using multiple sequences. Statistical science. 1992;7(4):457–472.

[14] Plummer M, Best N, Cowles K, Vines K. CODA: Convergence Diagnosis and Output Analysis for MCMC. R News. 2006;6(1):7–11. Available from: https://journal.r-project.org/archive/.

[15] Alkema L, Wong MB, Seah PR. Monitoring progress towards Millennium Development Goal 4: A call for improved validation of under-five mortality rate estimates. Statistics, Politics and Policy. 2012;3(2).

[16] Alkema L, Chao F, You D, Pedersen J, Sawyer CC. National, regional, and global sex ratios of infant, child, and under-5 mortality and identification of countries with outlying ratios: a systematic assessment. The Lancet Global Health. 2014;2(9):e521–e530.

[17] Chao F, You D, Pedersen J, Hug L, Alkema L. National and regional under-5 mortality rate by economic status for low-income and middle-income countries: a systematic assessment. The Lancet Global Health. 2018;6(5):e535–e547.

[18] Chao F, You D, Pedersen J, Hug L, Alkema L. Web appendix National and regional under-5 mortality rate by economic status for low-income and middle-income countries: a systematic assessment. 2018;DOI: 10.6084/m9.figshare.12442244. Available at https://ars.els-cdn.com/content/image/1-s2.0-S2214109X18300597-mmc1.pdf.
